# Diversity and composition of the microbiome associated with eggs of the Southern green stinkbug, *Nezara viridula* (Hemiptera: Pentatomidae)

**DOI:** 10.1002/mbo3.1337

**Published:** 2022-12-07

**Authors:** Margot W. J. Geerinck, Sara Van Hee, Gabriele Gloder, Sam Crauwels, Stefano Colazza, Hans Jacquemyn, Antonino Cusumano, Bart Lievens

**Affiliations:** ^1^ CMPG Laboratory for Process Microbial Ecology and Bioinspirational Management (PME&BIM), Department M2S, KU Leuven Leuven Belgium; ^2^ Leuven Plant Institute (LPI), KU Leuven Leuven Belgium; ^3^ Department of Agricultural, Food and Forest Sciences University of Palermo Viale delle Scienze Palermo Italy; ^4^ Interuniversity Center for Studies on Bioinspired Agro‐Environmental Technology (BATCenter) University of Napoli Federico II Portici Italy; ^5^ Laboratory of Plant Conservation and Population Biology, Biology Department, KU Leuven Leuven Belgium

**Keywords:** microbial community, *Pantoea*, Pentatomidae, *Sodalis*, symbiont

## Abstract

Although microbial communities of insects from larval to adult stage have been increasingly investigated in recent years, little is still known about the diversity and composition of egg‐associated microbiomes. In this study, we used high‐throughput amplicon sequencing and quantitative PCR to get a better understanding of the microbiome of insect eggs and how they are established using the Southern green stinkbug *Nezara viridula* (L.) (Hemiptera: Pentatomidae) as a study object. First, to determine the bacterial community composition, egg masses from two natural populations in Belgium and Italy were examined. Subsequently, microbial community establishment was assessed by studying stinkbug eggs of different ages obtained from laboratory strains (unlaid eggs collected from the ovaries, eggs less than 24 h old, and eggs collected 4 days after oviposition). Both the external and internal egg‐associated microbiomes were analyzed by investigating egg washes and surface‐sterilized washed eggs, respectively. Eggs from the ovaries were completely devoid of bacteria, indicating that egg‐associated bacteria were deposited on the eggs during or after oviposition. The bacterial diversity of deposited eggs was very low, with on average 6.1 zero‐radius operational taxonomic units (zOTUs) in the external microbiome and 1.2 zOTUs in internal samples of egg masses collected from the field. Bacterial community composition and density did not change significantly over time, suggesting limited bacterial growth. A *Pantoea*‐like symbiont previously found in the midgut of *N. viridula* was found in every sample and generally occurred at high relative and absolute densities, especially in the internal egg samples. Additionally, some eggs harbored a *Sodalis* symbiont, which has previously been found in the abdomen of several insects, but so far not in *N. viridula* populations. We conclude that the egg‐associated bacterial microbiome of *N. viridula* is species‐poor and dominated by a few symbionts, particularly the species‐specific obligate *Pantoea*‐like symbiont.

## Graphical abstract

In this study, we assessed the composition and establishment of the microbiome of insect eggs using the Southern green stinkbug *Nezara viridula* (Hemiptera: Pentatomidae) as a study object. Our results show that the egg‐associated bacterial microbiome is species‐poor and dominated by a few symbionts, particularly the species‐specific obligate *Pantoea*‐like symbiont.

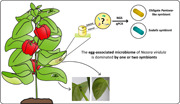

## 1 INTRODUCTION

Insects host a diversity of microbial communities in and on their bodies, and this microbiota can have a significant impact on host biology and development (Douglas, 2015; Hosokawa & Fukatsu, 2020). In recent years, several studies have focused on the microbiota from larval to adult stages (e.g., de Jonge et al., 2020; Xue et al., 2021), whereas relatively little is known about the microbiota associated with the earliest stages of life, that is, the egg (Nyholm, 2020). Egg microbiomes may host symbiotic microorganisms that protect developing embryos from invading pathogens or fouling (Flórez et al., 2015, 2017; Hilker et al., 2023). This can be especially important for insects that lay eggs in environments where eggs are exposed to high densities of microorganisms, such as soils or manure (Lam et al., 2009; Nyholm, 2020). Furthermore, egg‐associated microbiota can play a crucial role in embryological development or larval behavior, for example, by providing nutrients to developing embryos (Farine et al., 2017). Also, for some hosts, eggs serve as an excellent tool for the vertical transmission of essential symbionts between generations (Fukatsu & Hosokawa, 2002; Koga et al., 2012; Prado et al., 2006).

Examination of fresh eggs from the horn fly (*Haematobia irritans*; Diptera: Muscidae) has shown that the egg microbiome is dominated by the intracellular bacterial symbiont *Wolbachia*, reaching a relative abundance of 86% (Palavesam et al., 2012). *Wolbachia* is naturally present in a large number of insects and other arthropod species (Hilgenboecker et al., 2008). They are maternally transmitted across generations through the cytoplasm of eggs and confer a reproductive advantage to infected females through cytoplasmic incompatibility, feminization, male killing, or parthenogenesis (Stouthamer et al., 1999; Werren et al., 2008). Insect symbionts can also be transferred from parent to offspring by depositing the symbionts in capsules close to the eggs, in which they can survive the harsh conditions outside the host until they are acquired by newborn hatchlings (Fukatsu & Hosokawa, 2002). Likewise, in several insect families, gut symbionts are transferred via deposition of symbiont‐containing secretions from the anus on the eggs during oviposition (also known as “egg smearing”). The symbionts are then ingested by newly hatched nymphs, allowing the symbiont to pass through their digestive tract and establish in the crypts of the posterior midgut (Prado et al., 2006). Preventing newborns from orally acquiring symbionts seriously affects their fitness and survival (Tada et al., 2011). Many symbiotic gut bacteria possess the ability to contribute to essential traits such as defense mechanisms and nutrient acquisition, thereby providing important advantages to their hosts (Engel & Moran, 2013).

Although our understanding of the functional role of egg microbiota has increased substantially in recent years (Nyholm, 2020), surprisingly little is still known about the taxonomic composition and diversity of egg microbial communities. In this study, we used high‐throughput amplicon sequencing and targeted quantitative PCR (qPCR) to get a better understanding of insect egg microbiomes and how they are established and change over time using the Southern green stinkbug, *Nezara viridula* (L.) (Hemiptera: Pentatomidae), as the study object. This stinkbug species is widely distributed across (sub)tropical and Mediterranean regions of the world, where it causes damage to a broad range of important crops such as soybean and cotton. More recently, due to global warming, *N. viridula* has expanded its distribution range to north‐western Europe, where it attacks diverse vegetable crops, including tomato, sweet pepper, and cucumber (Conti et al., 2020). *N. viridula* females deposit usually 60–90 eggs in hexagonal clusters on the underside of leaves. In general, the first instars hatch after approximately 5 days (at 25°C), and five nymphal stages are completed before adulthood is reached (Esquivel et al., 2018). Following oviposition, the egg masses are “smeared” with a fecal secretion from the mother to vertically transmit beneficial symbionts to the offspring (Prado et al., 2006). This mode of symbiont transmission is well described in plant‐sucking stinkbugs (Pentatomidae) and parent bugs (Acanthosomatidae), but only very little is known about their entire egg microbiome. Here, we first examined bacterial diversity and taxonomic composition of the egg microbiome of *N. viridula* in samples from two natural populations from Belgium and Italy. Next, to examine how *N. viridula* egg microbial communities develop and evolve, a time‐series experiment was performed under laboratory conditions. Both external and internal microbiome samples were analyzed. Furthermore, for each sample, bacterial densities were quantified using qPCR.

## 2 MATERIALS AND METHODS

### 2.1 Sample collection

To assess the diversity and taxonomic composition of *N. viridula* egg‐associated bacterial communities, a number of egg masses were collected from two *N. viridula* populations (Table A1). Specifically, 15 egg masses (collected between August and September 2021) originated from a Belgian sweet pepper (*Capsicum annuum* L.; Solanaceae) greenhouse (Rijkevorsel, Belgium) infested with *N. viridula*. As it is difficult to find stinkbug eggs in a greenhouse, gravid females were caught in the greenhouse and placed on a mesh‐bagged sweet pepper leaf in the same greenhouse until oviposition. No insecticides were applied until at least 3 weeks before sample collection. Additionally, 15 egg masses were collected from a natural *Mirabilis jalapa* (Nyctaginaceae) population in Italy (Borgo Cavaliere, Palermo) between August and September 2020. All egg masses were collected using a pair of tweezers that was sterilized by applying 70% ethanol before the collection of each egg mass. Additionally, gloves were worn that were sterilized with 70% ethanol before an egg mass was collected. To ensure that egg masses were of comparable age, only white‐yellowish eggs were harvested, corresponding to an age of approximately 2 days. On average, egg masses contained 66 ± 8 (standard error) and 85 ± 4 eggs per egg mass for the Belgian and Italian stink bug populations, respectively. Immediately after collecting, egg masses were put individually in sterile 2 ml microcentrifuge tubes containing 1 ml of RNAlater (Sigma‐Aldrich) and brought to the laboratory. Samples were stored at −20°C until further processing.

To assess the temporal dynamics of the egg‐associated bacterial microbiome, a total of 70 egg samples were collected from two *N. viridula* laboratory strains (35 samples each) that were established with individuals collected in Belgium on the one hand and in Italy on the other hand (Table A1). The Belgian laboratory strain was reared and maintained on *C. annuum* plants (cv. “IDS RZ F1”; Rijk Zwaan) in insect cages (47.5 cm × 47.5 cm × 47.5 cm) (Bug‐Dorm‐4S4545; 114 MegaView Science Co. Ltd.) under controlled conditions (25 ± 1°C, 70 ± 2% relative humidity [RH] and a 16L:8D photoperiod), while the Italian laboratory strain was reared and maintained under similar conditions on *Vicia faba* plants (Fabaceae). Insects were fed with fresh organic vegetables (cherry tomatoes, white cabbage, haricots, and cauliflower) and organic seeds (sunflower, soybean, and peanut). Furthermore, water was provided as soaked cotton wool in a Petri dish. Food and water were renewed every 3 days. Newly laid eggs were collected daily to maintain the colonies. To avoid inbreeding, new field‐collected adults were regularly introduced into the colony. To obtain egg samples for our study, first unlaid eggs were collected from both laboratory strains (time point 0). Therefore, freshly killed gravid *N. viridula* females were dissected with the aid of a stereoscope (Olympus SZX12) under sterile conditions in a laminar flow cabinet, and mature eggs were harvested from the oviduct and pooled together to obtain five samples of 20 mature eggs per laboratory strain (5–10 eggs per female). Samples were put in RNAlater and stored at −20°C until further processing. Additionally, 15 freshly laid egg masses and 15 egg masses approximately 5 days old were sampled for each laboratory strain. To this end, five gravid *N. viridula* females were placed into a clean mesh insect cage (30 cm × 30 cm × 30 cm) (Vermandel) together with one *C. annuum* plant (cv. “IDS RZ F1”; Rijk Zwaan) and one *M. jalapa* plant for the Belgian and Italian laboratory strain, respectively. Plants were watered at need, and insects were provided tap water through wet cotton wool, while no additional food was provided. Cages were incubated under controlled conditions (23/21 ± 1°C L/D, 65 ± 2% RH, 16L:8D photoperiod) and monitored daily for egg deposition. Once egg masses were observed, stinkbugs were removed, and egg masses found on the leaves were collected either immediately (time point 1; less than 24 h old) or 4 days after oviposition (time point 2) until a total of 15 egg masses were obtained per laboratory strain per time point (Table A1). Egg masses were collected aseptically as mentioned above, and were individually put in 1 ml of RNAlater before storage at −20°C. Collected egg masses contained an average of 53 ± 4 and 69 ± 3 eggs per egg mass for the Belgian and Italian laboratory strains, respectively.

### 2.2 Microbiome sampling

Both the external and internal egg microbiomes were sampled. The external microbiota of the egg masses was obtained by vortexing the eggs in RNAlater for 1 min to enhance the detachment of associated microorganisms. Subsequently, the egg masses were removed using a sterilized pair of tweezers and placed in 2 ml microcentrifuge tubes containing 1 ml of sterile distilled water until further processing. The RNAlater solution containing the external microbiota was then centrifuged at 15,000*g* for 30 s, and the obtained cell pellet was resuspended in 1 ml lysis buffer for DNA extraction (buffer “CD1”; DNeasy PowerSoil Pro Kit; Qiagen). Subsequently, the entire volume was transferred into a 2 ml reaction tube with a screw cap (Greiner Bio‐One GmbH) containing a mixture of glass beads of different sizes (four beads of 3 mm in diameter and 200 µg of 150–212 µm glass beads) for further DNA extraction (see below). To obtain the internal microbiome, egg masses were taken out of the sterile distilled water, treated with 70% ethanol (10 min), then with 1.5% sodium hypochlorite (10 min), and finally washed four times with phosphate‐buffered saline with 0.01% Tween‐80 (Prado et al., 2006; Sare et al., 2020). The application of sodium hypochlorite is very effective in removing externally contaminating DNA (Binetruy et al., 2019; Greenstone et al., 2012). Next, surface‐sterilized egg masses were individually transferred into a 2 ml reaction tube with a screw cap containing 1 ml lysis buffer (”CD1”) and a mixture of glass beads as described above for sample crushing and further DNA extraction.

### 2.3 DNA extraction and molecular analysis

Genomic DNA was extracted from all samples using the DNeasy PowerSoil Pro Kit following the manufacturer's instructions with two modifications. First, 1 ml of lysis buffer “CD1” was used instead of 800 µl. Further, to homogenize the samples a Bead Ruptor Elite (Omni International) was used for two cycles at a speed of 5.5 m/s for 30 s (with a 30 s cooldown in between) instead of a vortex adapter. This way all egg samples were thoroughly ground and homogenized. In addition to the egg samples, two negative controls in which the sample was replaced by sterile, DNA‐free water (300 µl) were included to confirm the absence of reagent contamination. DNA samples were then subjected to PCR amplification of the hypervariable V4 region of the bacterial 16S ribosomal RNA (rRNA) gene using Illumina barcoded primers (primers 515F and 806R), designed according to Kozich et al. (2013) (Supporting Information: Table S1: https://doi.org/10.5281/zenodo.7326932). Two negative PCR controls (in which the DNA template was replaced by DNA‐free water) and one sample from a bacterial DNA mock community (Gloder et al., 2021) were included (Table A2). PCR amplification was performed in a 40 µl reaction volume, comprised of 2 µl template DNA, 0.5 µM of each primer, 150 µM of each dNTP, 1× Titanium *Taq* PCR buffer, and 1× Titanium *Taq* DNA polymerase (Takara Bio). The reactions were initiated by denaturation at 94°C for 120 s, followed by 35 cycles of 45 s at 95°C, 45 s at 59°C, 45 s at 72°C, and a final elongation step of 10 min at 72°C. Successful amplification of the samples was confirmed by 1.5% agarose gel electrophoresis. The negative DNA extraction and PCR controls showed no or very vague bands. Subsequently, purification of the PCR product was performed using Agencourt AMPure XP magnetic beads (Beckman Coulter Genomics GmbH) following the manufacturer's instructions. The concentration of the amplicons was measured with a Qubit high‐sensitivity fluorometer (Invitrogen), and samples were then pooled in equimolar concentrations. Next, following ethanol precipitation, the amplicon library was loaded onto a 1.5% agarose gel, and the target band was excised from the gel and purified using a QIAquick Gel Extraction Kit (Qiagen). The resulting library was diluted to 2 nM and sent for sequencing at the Center for Medical Genetics (University of Antwerp, Antwerp, Belgium). Sequencing was performed using an Illumina MiSeq sequencer with a v2 500‐Cycle Reagent Kit (Illumina).

Illumina sequences were received as a demultiplexed FASTQ file, with barcodes and primer sequences removed. Paired‐end reads were merged using USEARCH (v11.0.667) to form consensus sequences (Edgar, 2013) with no more than 10 mismatches allowed in the overlap region. Thereafter, sequences were truncated at the 250th base. Reads shorter than 250 bp or reads with a total expected error threshold above 0.1 were discarded using USEARCH (v11.0.667). Subsequently, Mothur (v1.39.5) commands “classify.seqs” and “remove.lineage” or “get.lineage” in combination with the SILVA database (v1.38) were used to identify and remove potential mitochondrial, chloroplast, or other nontarget sequences. Next, bacterial sequences were classified into zero‐radius operational taxonomic units (zOTUs) (Edgar, 2016), also known as amplicon sequence variants (ASVs) (Callahan et al., 2017) by the UNOISE3 algorithm as implemented in USEARCH (Edgar & Flyvbjerg, 2015). Further, the data set was analyzed in R (v3.5.2) using microDecon (v1.2.0) (McKnight et al., 2019) to correct for the presence of potential contaminants based on zOTU prevalence in the samples versus the mean of the PCR control samples (Davis et al., 2017; R Core Team, 2018). At the same time, the DNA extraction controls were removed from the data set since they yielded only very low sequence numbers and no additional zOTUs in comparison with the PCR controls. Next, before further processing, the data set was divided into two sub‐datasets, representing the data from the field‐collected egg masses on the one hand and the laboratory‐derived egg masses on the other hand. Furthermore, to eliminate potential contaminants, zOTUs occurring below a 1% relative abundance threshold per sample were removed from each data set. A cut‐off level of 1% has been shown to increase data accuracy, especially when microbial communities are composed of a small group of dominant organisms or to investigate microbiomes in low‐biomass 16S rRNA gene sequencing experiments (Díaz et al., 2021; Karstens et al., 2019). Moreover, zOTUs present in only one sample were eliminated. Finally, the number of sequences was rarefied to 2000 sequences per sample. The taxonomic origin of each zOTU was determined with the SINTAX algorithm as implemented in USEARCH based on the SILVA Living Tree Project v123. Further, the identity of the most important zOTUs was verified with a BLAST search in GenBank against type materials. The BLAST search was extended to the entire database when no significant similarity was found with type materials (<97% identity). Analysis of the mock communities demonstrated that only the taxa included in the mock were found (Supporting Information: Tables S2 and S3: https://doi.org/10.5281/zenodo.7326932), indicating that the experimental conditions were met to achieve robust data.

In all samples, the bacterial density was assessed through a qPCR assay using unmodified 515F/806R primers to determine the bacterial 16S rRNA gene copy numbers (for details, see Borremans et al., 2019). To quantify the *Pantoea*‐like symbiont abundantly found in our samples (see below), a qPCR analysis with the symbiont‐specific primers MMAOgroF/MMAOgroR, targeting a 140‐bp region of the chaperonin encoding *groEL* gene, was performed for all samples as described previously (Kikuchi et al., 2016). For each qPCR run, at least two negative controls were included. All qPCR assays were performed in duplicate and a *C*_T_ value of 35 was taken as the detection threshold, which was below the *C*_T_ value obtained for any negative control sample.

### 2.4 Statistical analyses

To determine whether both data sets covered the expected microbial diversity, rarefaction curves were generated using the Phyloseq package in R showing the number of observed zOTUs as a function of the number of sequences (McMurdie & Holmes, 2013; R Core Team, 2018). A Mann–Whitney *U*‐test was performed to determine whether zOTU richness and bacterial density were affected by sample origin, that is, external versus internal samples. For the laboratory‐collected eggs, a Kruskal‐Wallis rank‐sum test was carried out as well to assess whether age affected zOTU richness and bacterial density. In addition, a Dunn's test with Benjamini–Hochberg correction was performed for multiple pairwise comparisons. For statistical analysis of the qPCR results, samples that did not exceed the detection limit were assigned the gene copy number corresponding to the qPCR detection threshold, that is, 3.1 × 10^3^ 16S rRNA and 1.1 × 10^3^ *groEL* gene copies per egg mass.

## 3 RESULTS

### 3.1 Diversity and taxonomic composition of egg‐associated bacterial communities in natural *N. viridula* populations

**Figure 1:**
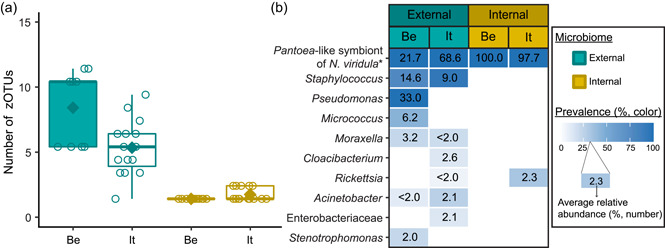
Zero‐radius operational taxonomic unit (zOTU) richness (a) and composition (b) of egg‐associated bacterial communities (external and internal microbiome samples) from natural *Nezara viridula* populations sampled in Belgium (Be) and Italy (It). The upper and lower whiskers of the boxplots correspond to the first and third quartiles, while the bar in bold represents the median and the diamond the average per subgroup. The value of each data point is indicated by a dot. The bacteria in (b) represent the most prevalent taxa in the different subgroups (present at a mean relative abundance ≥2.0% in at least one subgroup). For each zOTU, the mean relative abundance for each subgroup is given in the box as a percentage, whereas the color indicates prevalence (white is absent). zOTUs are identified by a BLAST search against type materials in GenBank. When no significant similarity was found, the analysis was performed against the entire GenBank (indicated with an asterisk). Identifications were performed at the genus level; when identical scores were obtained for different genera, identifications were performed at the family level.

**Figure A1:**
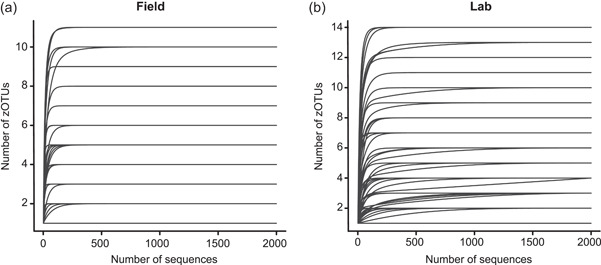
Rarefaction curves for the different samples studied. Egg masses were collected from natural (a) and laboratory‐reared *Nezara viridula* populations (b). Rarefaction curves approached saturation, indicating that our sequencing depth was sufficient to cover the microbial diversity.

After quality filtering, removal of low abundant zOTUs, and rarefaction to 2000 sequences per sample, a total of 54 samples and 37 bacterial zOTUs were retained for further analysis (Supporting Information: Table S2: https://doi.org/10.5281/zenodo.7326932). Six external samples from the Belgian population were removed from the data set since they yielded too low sequence numbers for further analysis. Rarefaction curves approached saturation, implying that a sequence depth of 2000 sequences was adequate to cover the bacterial diversity (Figure A1a). The internal microbiome samples contained on average 1.2 bacterial zOTUs (Figure 1a). All internal samples from the Belgian population contained one zOTU; for the Italian population, the number of zOTUs varied between one and two (Table A3). The external microbiome was significantly more diverse than the internal microbiome (*W*_1_ = 17.5; *p* < 0.001) (Table A4), with an average of 8.0 (range: 5–11) and 4.9 zOTUs (range: 1–9) per sample for the Belgian and Italian population, respectively (Figure 1a and Table A3). In all samples, a *Pantoea*‐like symbiont previously identified in *N. viridula* (zOTU1) was found (Figure 1b and Supporting Information: Table S2: https://doi.org/10.5281/zenodo.7326932). While the internal samples of the Belgian population only contained this symbiont, a *Rickettsia* species (zOTU10) was also present in 33.3% of the Italian samples (mean relative abundance: 2.3%). Hence, a relative abundance of the *Pantoea*‐like symbiont ranged from 85.3% to 100% (average: 97.7%) in the internal samples from the Italian *N. viridula* population (Figure 1b). Similarly, the *Pantoea*‐like symbiont was consistently found in the external samples, albeit in lower relative abundance. For the Italian population, the symbiont occurred at a mean relative abundance of 68.6% in the external samples (range: 18.2%–100%), while this was 21.7% (range: up to 58.1%) for the Belgian population. Additionally, the external samples contained a number of environmental and insect‐associated bacteria. For example, *Staphylococcus* sp. (zOTU12) was present at a mean relative abundance of 14.6% and 9.0% in the external samples of the Belgian and Italian stinkbug populations, respectively, while *Pseudomonas* sp. (zOTU34) was exclusively present in external samples from the Belgian population. Moreover, in these samples, this zOTU was found in every sample and occurred at a mean relative abundance of 33.0% (range: 6.6%–85.7%) (Figure 1b and Supporting Information: Table S2: https://doi.org/10.5281/zenodo.7326932).

**Figure 2:**
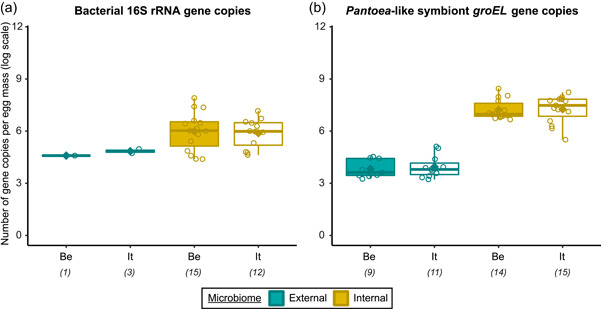
Density of egg‐associated bacterial communities (external and internal microbiome samples) in natural *Nezara viridula* populations, sampled in Belgium (Be) and Italy (It). Boxplots show the number of bacterial 16S ribosomal RNA (rRNA) gene copies (a) and *groEL* gene copies of the *Pantoea*‐like symbiont (b) per egg mass. The upper and lower whiskers correspond to the first and third quartiles, while the bar in bold represents the median and the diamond the average per subgroup. The value of each data point is indicated by a dot. The number of positive samples included in the analysis is presented under the *x*‐axis between brackets. The limits of detection were 3.1 × 10^3^ 16S rRNA (a) and 1.1 × 10^3^ *groEL* (b) gene copies per egg mass.

In general, bacterial density was low in the external samples (Figure 2a). For the Belgian population, 16S rRNA gene copy numbers did not exceed the detection threshold of 3.1 × 10^3^ gene copies per egg mass (corresponding to a *C*_T_ value of 35) in 14 out of 15 samples. Likewise, in 12 out of 15 samples of the Italian population, 16S rRNA gene copy numbers remained below the detection threshold. In contrast, the internal samples contained on average 9.6 × 10^6^ and 2.6 × 10^6^ 16S rRNA gene copy numbers per egg mass for the Belgian and Italian population, respectively (samples below the detection threshold excluded, i.e., three Italian samples) (Figure 2a and Table A5). *groEL* gene copy numbers of the *Pantoea*‐like symbiont were significantly different between the external and internal samples (*W*_1_ = 875.0; *p* < 0.001) (Figure 2b and Table A4). The external samples of the Belgian and Italian population contained on average 1.2 × 10^4^ and 2.7 × 10^4^ *PantoeagroEL* gene copies per egg mass, respectively (samples below the detection limit of 1.1 × 10^3^ *groEL* gene copies per egg mass excluded, i.e., six Belgian and four Italian samples). For the internal samples, copy numbers in both populations reached an average of 4.5 × 10^7^ per egg mass (samples below the detection limit excluded, i.e., one Belgian sample).

### 3.2 Temporal dynamics in the diversity and composition of egg‐associated bacterial communities in *N. viridula*

**Figure 3:**
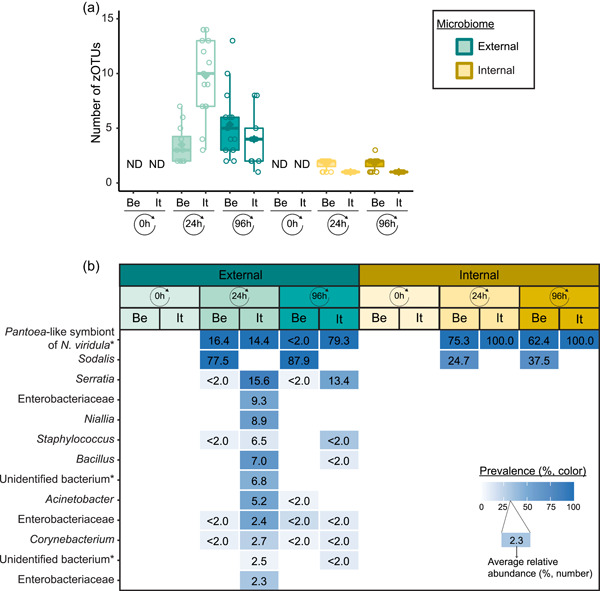
Temporal dynamics in zero‐radius operational taxonomic unit (zOTU) richness (a) and composition (b) of egg‐associated bacterial communities from *Nezara viridula* laboratory strains obtained with insects from Belgium (Be) and Italy (It). The upper and lower whiskers of the boxplots correspond to the first and third quartiles, while the bar in bold represents the median and the diamond the average per subgroup. The value of each data point is indicated by a dot, while “ND” refers to “no bacteria detected.” The bacteria in panel b represent the most prevalent taxa in the different subgroups (present at a mean relative abundance ≥2.0% in at least one subgroup). For each zOTU, the mean relative abundance for each subgroup is given in the box as a percentage, whereas the color indicates prevalence (white is absent). zOTUs are identified by a BLAST search against type materials in GenBank. When no significant similarity was found, the analysis was performed against the entire GenBank (indicated with an asterisk). Identifications were performed at the genus level; when identical scores were obtained for different genera, identifications were performed at the family level. Hits with uncultured bacteria are indicated as “unidentified bacterium.”

Following PCR amplification and amplicon sequencing, nine samples were removed from the data set due to low sequence numbers, that is, three external samples from freshly laid eggs from the Belgian laboratory strain and six external samples of 4‐day‐old egg masses from the Italian laboratory strain. Likewise, no bacteria were detected in the unlaid eggs. Bioinformatics analysis revealed a total of 50 bacterial zOTUs (Supporting Information: Table S3: https://doi.org/10.5281/zenodo.7326932) and rarefaction curves approached saturation (Figure A1b). The internal microbiome of deposited eggs contained on average 1.4 zOTUs (time points 1 and 2 combined) (Figure 3a and Table A6). All internal samples of the Italian laboratory strain contained one zOTU; for the Belgian laboratory strain, the number of zOTUs varied between one and three. Bacterial zOTU richness in the external samples (Figure 3a) was significantly higher (*W*_1_ = 809.0; *p* < 0.001) (Table A7). For freshly laid egg masses, the average number of zOTUs in the external samples was 3.5 (range: 2–7) and 9.8 (range: 3–14) for the Belgian and Italian laboratory strains, respectively. In external samples from older eggs, the average number of zOTUs was 5.3 (range: 2–13) and 4.0 (range: 1–8) for the Belgian and Italian strains, respectively. The number of zOTUs did not change significantly after egg deposition between freshly laid egg masses and 4‐day‐old egg masses (Dunn's test, Z_2_ = 0.74, *p* = 0.46).

All samples with exception of the unlaid eggs contained the *Pantoea*‐like symbiont (zOTU1; Figure 3b and Supporting Information: Table S3: https://doi.org/10.5281/zenodo.7326932). This symbiont was the only bacterium detected in the internal egg samples from the Italian laboratory strain, while internal egg samples from the Belgian laboratory strain also harbored a bacterial species from the genus *Sodalis* (zOTU2), which was present in 73.3% of the samples (unlaid eggs excluded) (Figure 3b). In these eggs, the *Pantoea*‐like symbiont occurred at a mean relative abundance of 75.3% (range: 33.7%–100%) in freshly deposited egg masses and slightly decreased to 62.4% (range: up to 100%) after 4 days (Figure 3b). Similar results were observed for the external samples. In contrast to the internal samples, *Sodalis* dominated the microbiome in the external samples of the Belgian laboratory strain, accounting for a mean relative abundance of 77.5% (range: 49.1%–98.8%) in freshly deposited egg masses and 87.9% (range: 59.2%–99.5%) after 4 days. In these samples, the *Pantoea*‐like symbiont occurred at a mean relative abundance of 16.4% (range: up to 51.0%) and 1.2% (range: up to 6.1%), respectively. In the external samples of the Italian laboratory strain, the symbiont was present at a mean relative abundance of 14.4% (range: up to 70.3%) and 79.3% (range: 50.5%–100%) in freshly laid eggs and 4‐day‐old eggs, respectively (Figure 3b). In addition, the external samples contained a number of environmental and insect‐associated bacteria, especially egg masses less than a day old from the Italian laboratory strain (Figure 3b).

**Figure 4:**
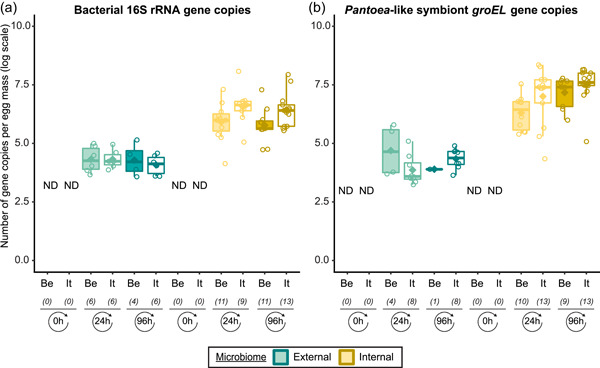
Density of egg‐associated bacterial communities (external and internal microbiome samples) from *Nezara viridula* laboratory strains, obtained with insects from Belgium (Be) and Italy (It). Boxplots show the number of bacterial 16S rRNA gene copies (a) and *groEL* gene copies of the *Pantoea*‐like symbiont (b) per egg mass. The upper and lower whiskers correspond to the first and third quartiles, while the bar in bold represents the median and the diamond the average per subgroup. The value of each data point is indicated by a dot, while “ND” refers to “no bacteria detected.” The number of positive samples included in the analysis is presented under the *x*‐axis between brackets. The limits of detection were 3.1 × 10^3^ 16S rRNA (a) and 1.1 × 10^3^ *groEL* (b) gene copies per egg mass.

In general, bacterial density assessed by qPCR was low in external samples (Figure 4a). None of the egg samples dissected from the ovaries exceeded the qPCR detection threshold. For egg masses less than 24 h old, 16S rRNA gene copy numbers did not exceed the detection threshold of 3.1 × 10^3^ gene copies per egg mass in 9 out of 15 samples for both laboratory strains. Similarly, 11 and 9 out of 15 external samples of older egg masses remained below the detection limit for the Belgian and Italian laboratory strains, respectively (Table A8). By contrast, internal samples contained a significantly higher number of 16S rRNA gene copies compared to the external samples (*W*_1_ = 3683.0; *p* < 0.001) (Table A7), and a total of 44 out of 60 samples (unlaid eggs excluded) exceeded the detection threshold (Table A8). For freshly laid egg masses, internal samples contained on average 3.0 × 10^6^ and 1.7 × 10^7^ 16S rRNA gene copy numbers per egg mass for the Belgian and Italian laboratory strains, respectively (samples below the detection threshold excluded, that is, four Belgian and six Italian samples). Similarly, internal samples of older egg masses contained on average 2.4 × 10^6^ and 1.2 × 10^7^ 16S rRNA gene copy numbers per egg mass, respectively (samples below the detection threshold excluded, i.e., four Belgian and two Italian samples) (Figure 4a and Table A8). Bacterial densities did not change significantly between freshly laid eggs and 4‐day‐old egg masses (Dunn's test, *Z*_2_ = −0.34, *p* = 0.73). A similar trend was observed for the number of *groEL* gene copies of the *Pantoea*‐like symbiont (Figure 4b). For egg masses dissected from the ovaries, none of the samples exceeded the detection threshold. The number of *PantoeagroEL* gene copies was low in the external samples of the deposited eggs for both laboratory strains. In contrast, the *groEL* gene copy number was significantly higher in the internal samples (*W*_1_ = 3741.5; *p* < 0.001) (Table A7). Furthermore, symbiont densities did not change significantly over time between freshly laid egg masses and 4‐day‐old egg masses (Dunn's test, *Z*_2_ = −0.05, *p* = 0.96) (Figure 4b).

## 4 DISCUSSION

Although the microbiome of insects has been studied increasingly in recent years (e. g., Gloder et al., 2021; de Jonge et al., 2020; Xue et al., 2021), so far only little attention has been given to the microbiome of insect eggs (Nyholm, 2020). Our taxonomic analysis of the egg‐associated microbiome of *N. viridula* revealed that bacterial diversity was low. On average 6.1 zOTUs were found on the eggs of natural *N. viridula* populations, while on average only 1.2 zOTU was associated with the internal samples. Similarly, low microbial diversity has been found in the midgut of field‐collected *N. viridula* adults (Medina et al., 2018), suggesting that overall microbial diversity associated with *N. viridula* is low: no culturable bacteria were found in the V1–V3 midgut sections in more than 54% of *N. viridula* adults collected in the field, while the rest of the stinkbugs were colonized by only a few culturable bacteria like *Bacillus*, *Enterococcus*, *Micrococcus*, *Pantoea*, *Staphylococcus*, and *Yokenella* (Medina et al., 2018).

The obligate *Pantoea*‐like symbiont of *N. viridula* (zOTU1) was found in all samples investigated (except in unlaid eggs). However, in laboratory samples, its relative abundance was lower than in samples from natural populations. This *Pantoea*‐like symbiont inhabits the crypts of the posterior section of the midgut in *N. viridula* (Hirose et al., 2006; Prado et al., 2006; Tada et al., 2011), and is known to be transferred via egg smearing to the next generation of stinkbugs (Prado et al., 2006). Previous research has shown that removal of the symbiont by egg surface sterilization or heat causes severe fitness defects in emerged nymphs, including retarded nymphal growth and lower nymphal survival (Kikuchi et al., 2016; Tada et al., 2011). Nevertheless, a decrease in fitness was not found in a study on a Hawaiian *N. viridula* population (Prado et al., 2006), suggesting that other factors such as food resources and environmental and/or genetic factors can influence the performance of stinkbug populations (Prado et al., 2006, 2009). Several pentatomid stinkbug species harbor a species‐specific obligate symbiont belonging to the *Pantoea* genus that resides in symbiotic midgut crypts. These symbionts act as mutualists, but their effects on host fitness remain elusive (Duron & Noël, 2016). Typically, they harbor reduced genomes, which suggests an evolution‐driven specialization of their interaction with their host (Hosokawa et al., 2016; Kashkouli et al., 2021). Strikingly, the highest densities of the symbiont were found in the internal samples, suggesting that the *Pantoea*‐like symbiont is tightly associated with the eggshell (and therefore could not be removed by washing) and/or resides in the eggshell pores or the interior of the eggs enhancing protection from environmental hazards. So far, it cannot be excluded that our “internal” samples represent bacteria in or on the eggshell that could not be detached by washing, rather than microorganisms occurring in the interior of the eggs. The symbiont might migrate into the eggshell or inside the eggs through passive penetration via micropyles, that is, tube‐like hollow protrusions of the chorion (Esselbaugh, 1946). This mechanism has been reported for the human head lice *Pediculus humanus capitis*, where the uptake of its primary endosymbiont belonging to the family of *Enterobacteriaceae* is facilitated by hydropyles in the eggshell of the oocyte (Perotti et al., 2007).

Eggs from the Belgian laboratory strain harbored a second symbiont belonging to the genus *Sodalis* in both the investigated external and internal samples, while the bacterium was absent in egg masses from the natural populations and the Italian laboratory strain. Instead, the external microbiome of the Italian laboratory strain contained several other bacteria that were not found on egg masses of the Belgian laboratory strain and were also absent from eggs of the corresponding natural population. These differences could be due to differences in the genotype of the stinkbug or the different plant species from which eggs were collected. Differences in microbiome structure between natural and laboratory‐reared insect populations have been observed frequently (e.g., Chandler et al., 2011; Gloder et al., 2021; Park et al., 2019), and seem to be driven by diverse factors such as rearing conditions and rearing environment, habitat, and diet (Engel & Moran, 2013; Medina et al., 2022; Wang et al., 2019; Yun et al., 2014). Furthermore, it has to be noted that the Belgian laboratory‐reared population was not derived from the same geographical location as the Belgian natural population. *Sodalis* symbionts have been found among multiple insects including several stinkbug species. However, to the best of our knowledge, *Sodalis* symbionts have not been reported in *N. viridula*. The association is most likely facultative due to the overall host–symbiont phylogenetic incongruence and relatively low infection frequencies (Hosokawa et al., 2015). In the lygaeoid bug *Henestaris halophilus* (Heteroptera: Henestarinae), the *Sodalis* symbiont is characterized as a mutualistic endosymbiont providing its host with amino acids and cofactors. Moreover, it is believed that reductive genome evolution is ongoing, strengthening its symbiotic relationship (Santos‐Garcia et al., 2017). No bacteria were detected in eggs dissected from the ovaries, indicating that all bacteria found originated from post‐oviposition processes, such as egg smearing, inoculation by the air, or from the plant. Further, bacterial densities as well as microbial community composition did not change significantly over time, suggesting that the eggs do not or only weakly support bacterial growth by providing only a few nutrients, a strategy that may particularly protect the eggs from fouling or pathogen invasion. Whether this is truly the case for *N. viridula* remains to be investigated.

## 5 CONCLUSIONS

Altogether, our results show that the diversity of the egg‐associated bacterial microbiome of *N. viridula* was very low, and dominated by a few bacteria. Further, we showed that the egg microbiome did not change significantly over time. A *Pantoea*‐like symbiont previously found in the midgut of *N. viridula* was found in every sample investigated and generally occurred at high relative and absolute densities, especially in samples representing the eggshell and the interior of the eggs. In addition, a *Sodalis* symbiont was found in eggs from the Belgian laboratory strain, which was not found in the other investigated populations. Further research is needed to unravel the functional role of this bacterium.

## AUTHOR CONTRIBUTIONS

**Margot W. J. Geerinck**: Data curation (lead); formal analysis (lead); investigation (lead); methodology (lead); visualization (lead); writing – original draft (lead); writing – review and editing (equal). **Sara Van Hee**: Methodology (supporting); writing – review and editing (supporting). **Gabriele Gloder**: Methodology (supporting); writing – review and editing (supporting). **Sam Crauwels**: Formal analysis (supporting); software (lead); writing – review and editing (supporting). **Stefano Colazza**: Conceptualization (equal); investigation (supporting); writing – review and editing (equal). **Hans Jacquemyn**: Conceptualization (equal); formal analysis (supporting); investigation (supporting); visualization (supporting); writing – original draft (supporting); writing – review and editing (equal). **Antonino Cusumano**: Conceptualization (equal); data curation (supporting); investigation (supporting); methodology (supporting); visualization (supporting); writing – original draft (supporting); writing – review and editing (equal). **Bart Lievens**: Conceptualization (lead); formal analysis (supporting); funding acquisition (lead); supervision (lead); visualization (supporting); writing – original draft (supporting); writing – review and editing (equal).

## CONFLICT OF INTEREST

None declared.

## ETHICS STATEMENT

None required.

## Supporting information

Supporting information.

## Data Availability

The sequences obtained in this study were deposited in the Sequence Read Archive (SRA) at NCBI under BioProject PRJNA869923 (accession numbers SAMN30338702–SAMN30338764 and SAMN30369493–SAMN30369650): https://www.ncbi.nlm.nih.gov/bioproject/PRJNA869923. Further, underlying experimental data (Supporting Information: Table S1–S3) can be found in the Zenodo repository at https://doi.org/10.5281/zenodo.7326932 (Supporting Information: Table S1: Primer design and sample‐specific barcodes; Table S2: Identification of bacterial zero radius operational taxonomic units [zOTUs] according to the Silva v1.23 database and distribution over the investigated samples for field‐collected egg masses; Table S3: Identification of bacterial zOTUs according to the Silva v1.23 database and distribution over the investigated samples for egg masses obtained with the laboratory‐reared populations).

## Appendix

See Tables A1, A2, A3, A4, A5, A6, A7, A8.

**Table A1:** Sample details

Sample ID	Origin	GPS coordinates	Isolation source	Age eggs	Number of samples	
					External (E)	Internal (I)
FBe < “Microbiome”> xx	Field (F), Belgium (Be)	51°20′18.7″ N 4°44′34.9″ E	*Capsicum annuum* L.	ca. 2 days	15	15
FIt < “Microbiome”> xx	Field (F), Italy (It)	37°44′22.6″ N 13°08′27.4″ E	*Mirabilis jalapa*	ca. 2 days	15	15
L0Be < “Microbiome”> xx	Lab (L), KU Leuven, Belgium (Be)		None, dissected from the oviduct	Unlaid (L0)	5	5
L0It < “Microbiome”> xx	Lab (L), SAAF, Italy (It)		None, dissected from the oviduct	Unlaid (L0)	5	5
L1Be < “Microbiome”> xx	Lab (L), KU Leuven, Belgium (Be)		*Capsicum annuum* L. cv. IDS RZ F1	<24 h (L1)	15	15
L1It < “Microbiome”> xx	Lab (L), SAAF, Italy (It)		*Mirabilis jalapa*	<24 h (L1)	15	15
L2Be < “Microbiome”> xx	Lab (L), KU Leuven, Belgium (Be)		*Capsicum annuum* L. cv. IDS RZ F1	ca. 4 days (L2)	15	15
L2It < “Microbiome”> xx	Lab (L), SAAF, Italy (It)		*Mirabilis jalapa*	ca. 4 days (L2)	15	15

Abbreviation: GPS, Global Positioning System.

**Table A2:** Composition of the mock community

Composition	Species
Organism 1	*Acinetobacter apis*
Organism 2	*Acinetobacter nectaris*
Organism 3	*Asaia* sp.
Organism 4	*Cronobacter sakazakii*
Organism 5	*Pseudomonas* sp.
Organism 6	*Pseudomonas syringae*
Organism 7	*Stenotrophomonas* sp.

**Table A3:** Diversity metrics for the bacterial communities of the field‐collected egg masses

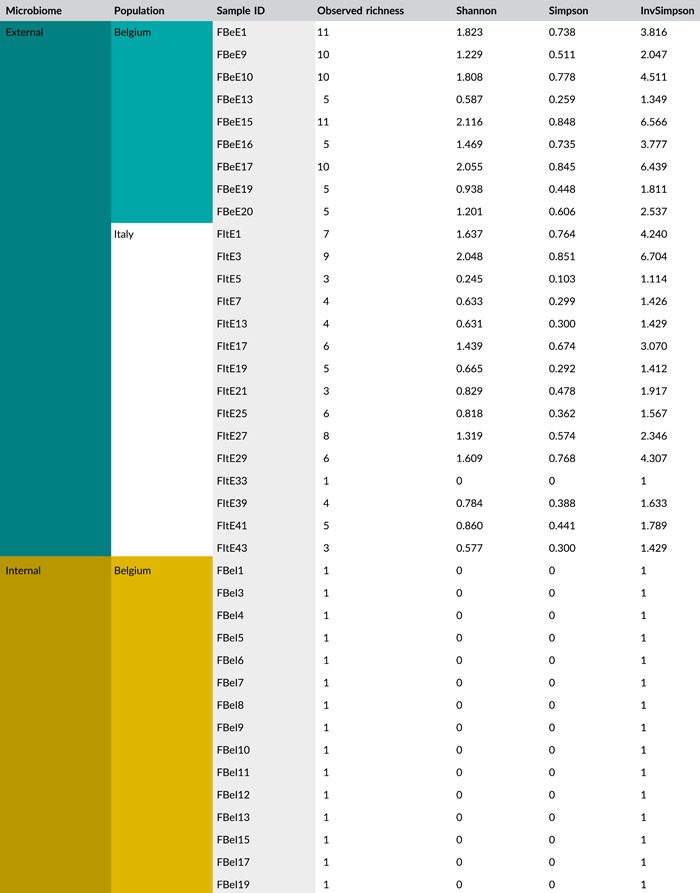

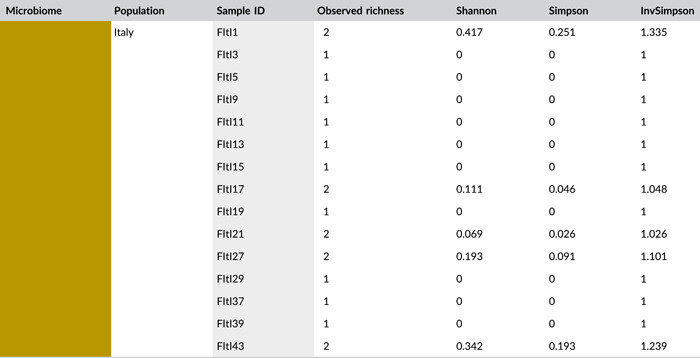

**Table A4:** Results of Mann–Whitney *U*‐test on observed zOTU richness, densities for bacteria, and the *Pantoea*‐like symbiont for field‐collected egg masses

Parameters	Observed richness		Bacterial density^a^		Symbiont density^b^	
	*W*	*p* Value	*W*	*p* Value	*W*	*p* Value
Microbiome	17.50	<0.001	829.00	<0.001	875.00	<0.001

Abbreviations: rRNA, ribosomal RNA; zOTU, zero‐radius operational taxonomic unit.

^a^ Determined as 16S rRNA gene copy numbers.

^b^ Determined as *groEL* gene copy numbers of the *Pantoea*‐like symbiont.

**Table A5:** Determination of bacterial 16S rRNA gene copy numbers and *groEL* gene copy numbers of the *Pantoea*‐like symbiont for the field‐collected egg masses using qPCR

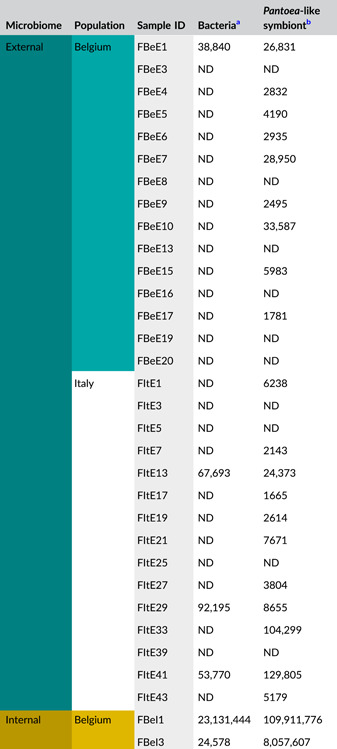

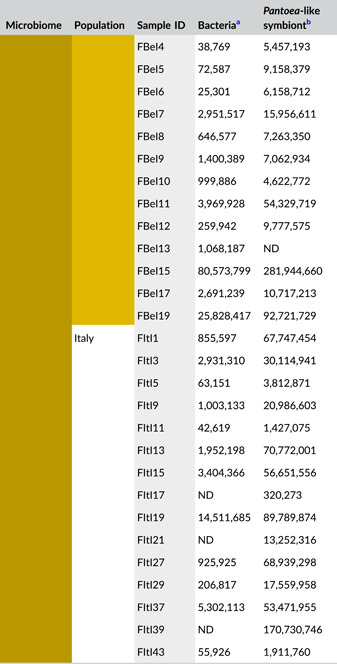

Abbreviations: ND, not detected (below detection limit); qPCR, quantitative polymerase chain reaction; rRNA, ribosomal RNA; zOTU, zero‐radius operational taxonomic unit.

^a^ Determined as 16S rRNA gene copy numbers per egg mass.

^b^ Determined as *groEL* gene copy numbers of the *Pantoea*‐like symbiont per egg mass.

**Table A6:** Diversity metrics for the bacterial communities of the egg masses obtained with the laboratory‐reared populations

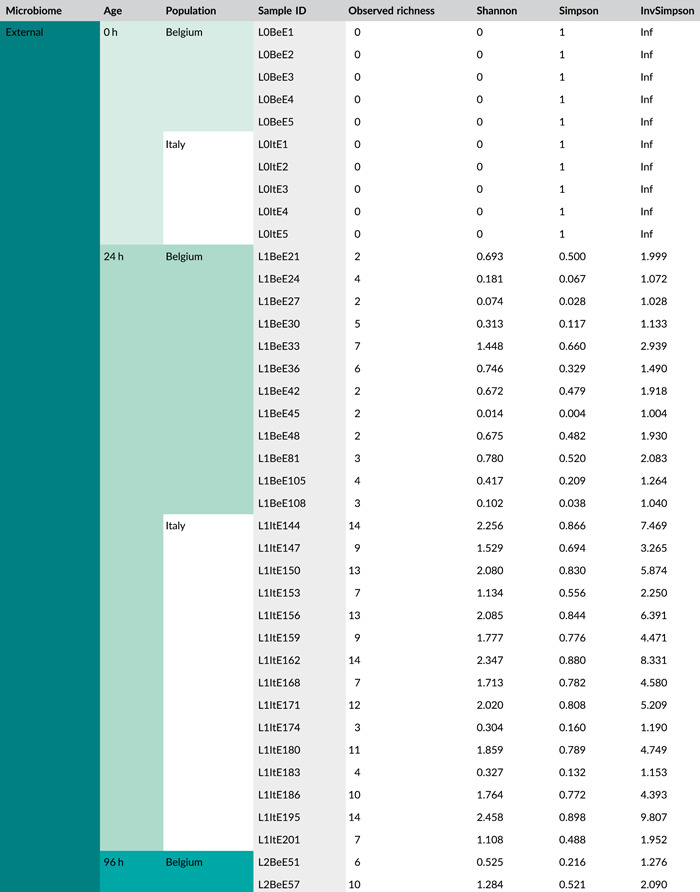

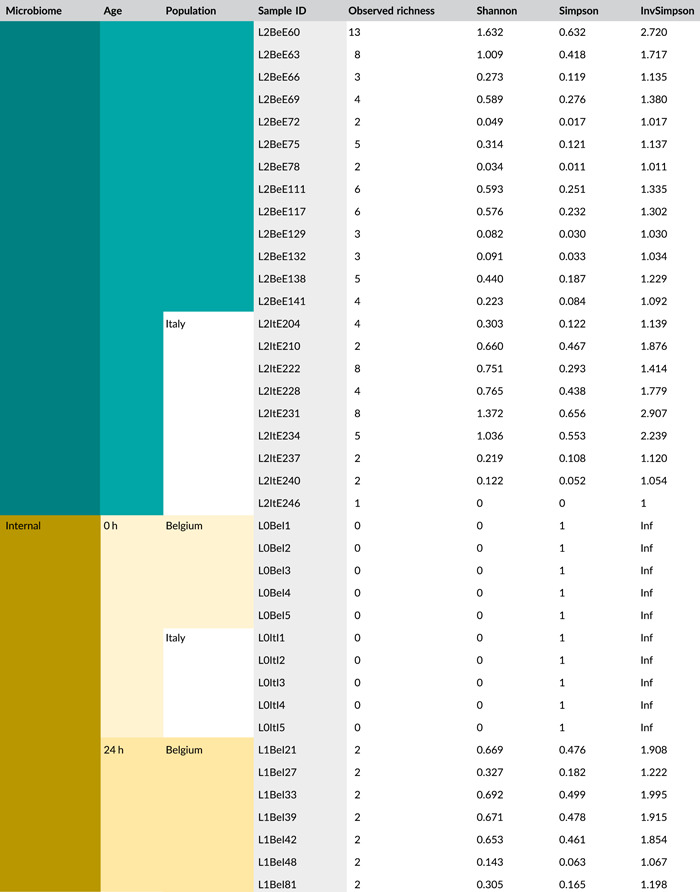

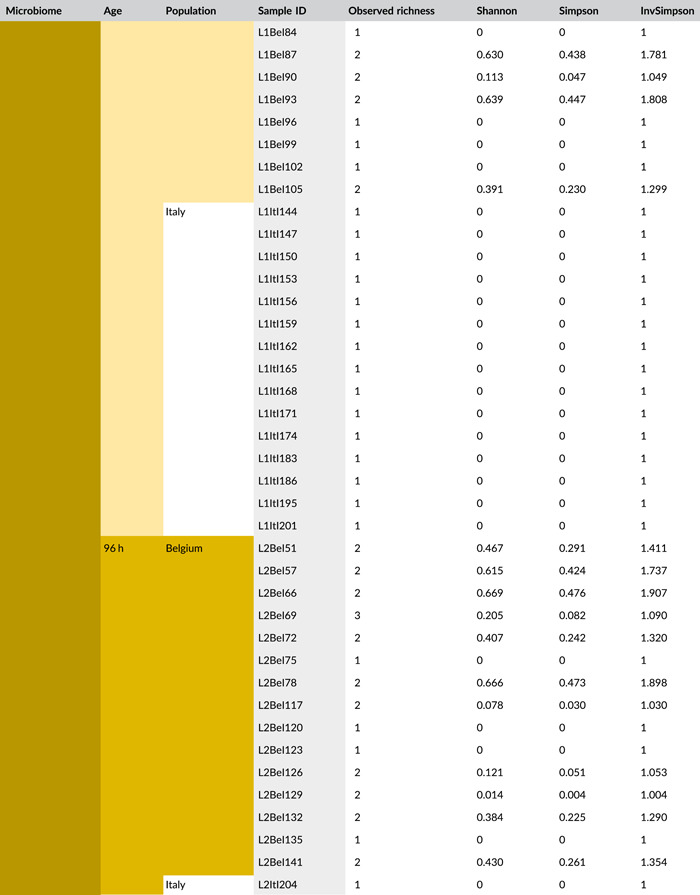

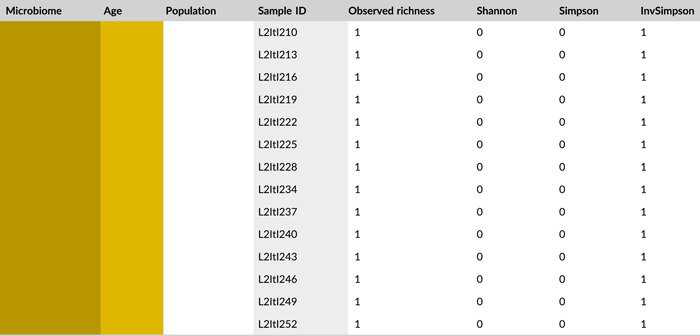

**Table A7:** Results of Mann–Whitney *U*‐test and Kruskal–Wallis rank‐sum test on observed richness, densities for bacteria, and the *Pantoea*‐like symbiont for egg masses obtained with the laboratory‐reared populations

Parameters	Observed richness		Bacterial density		Symbiont density	
	*W*	*p* Value	*W*	*p* Value	*W*	*p* Value
Microbiome	809.00	<0.001	3683.00	<0.001	3741.50	<0.001

**Parameters**	**Observed richness**		**Bacterial density**		**Symbiont density**	
	***X*** **^2^**	***p*** Value	***X*** **^2^**	***p*** Value	***X*** **^2^**	***p*** Value
Age	53.30	<0.001	18.24	<0.001	18.13	<0.001

Abbreviation: rRNA, ribosomal RNA.

^a^Determined as 16S rRNA gene copy numbers.

^b^Determined as *groEL* gene copy numbers of the *Pantoea*‐like symbiont.

**Table A8:** Determination of bacterial 16S rRNA gene copy numbers and *groEL* gene copy numbers of the *Pantoea*‐like symbiont for egg masses obtained with the laboratory‐reared populations using qPCR

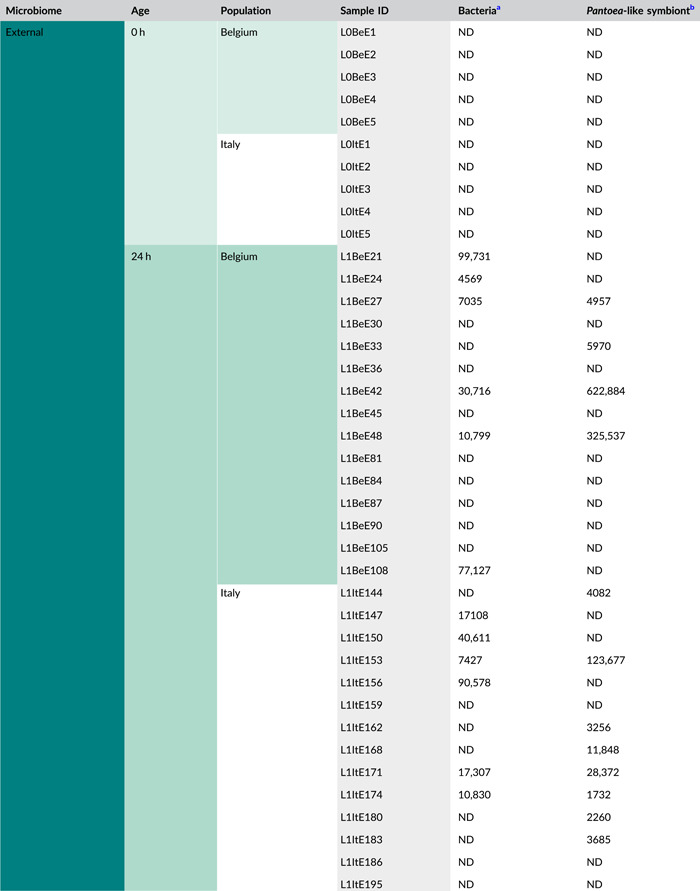

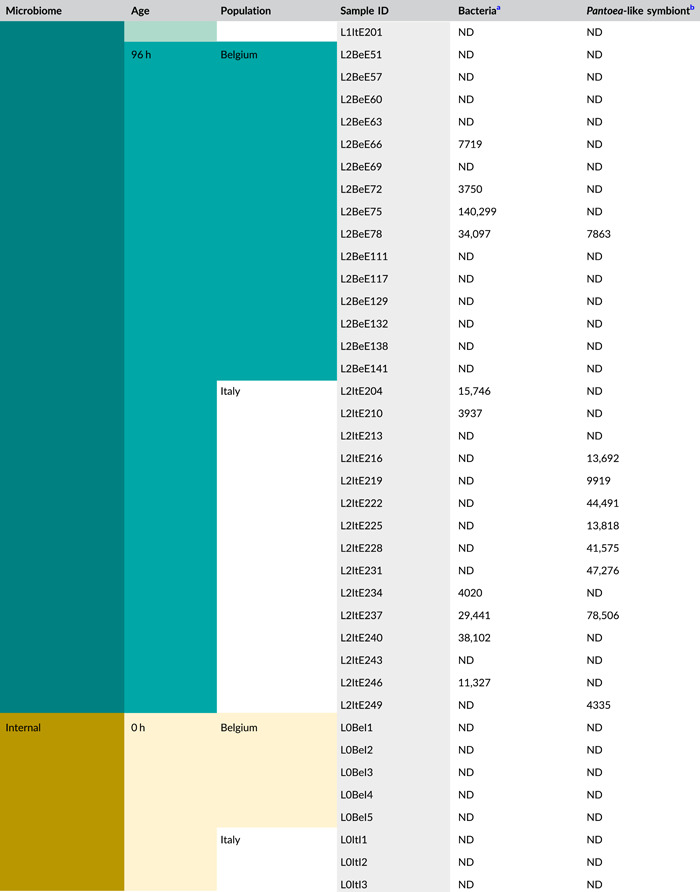

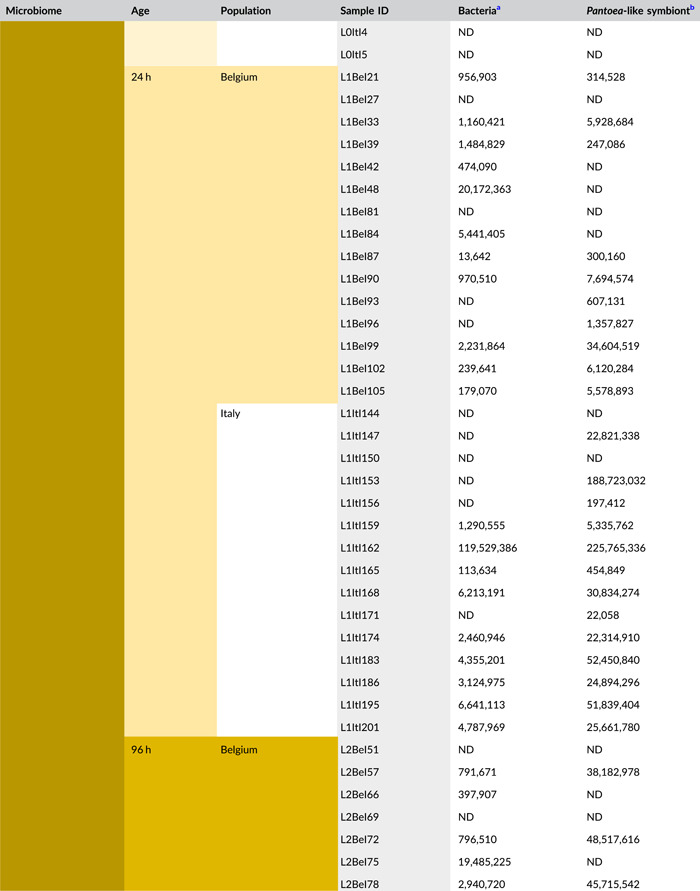

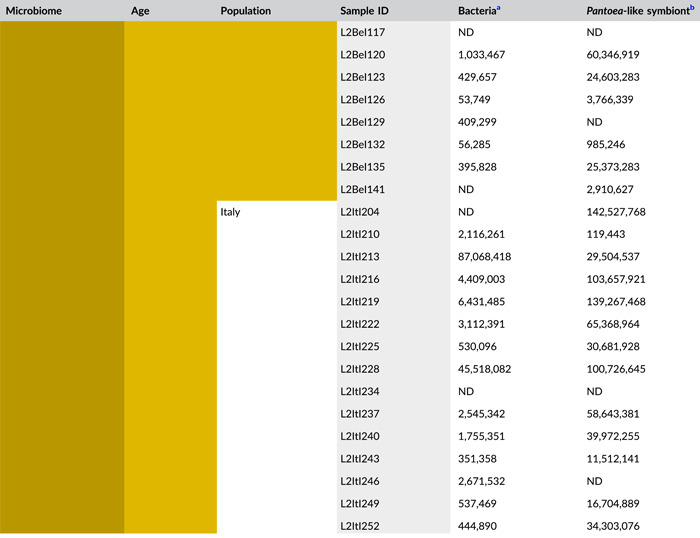

Abbreviations: ND, not detected (below detection limit); qPCR, quantitative polymerase chain reaction; rRNA, ribosomal RNA.

^a^ Determined as 16S rRNA gene copy numbers per egg mass.

^b^ Determined as *groEL* gene copy numbers of the *Pantoea*‐like symbiont per egg mass.
